# Miscellaneous Items

**Published:** 1856-06

**Authors:** 


### Miscellaneous Items.

Dr. Roberts described, before the Royal Scottish Society of Arts, his method for cauterizing the dental nerve, whereby a tooth may be stopped without pain, or a stump become a support for a new tooth; while the use of arsenic, and the ordinary intimidating mode of cauterization, are avoided. He applies a wire to the patient's tooth, and heats it by means of a small Grove's battery. "The advantages," he says, "to be obtained by this instrument are: its easy application to the desired spot in the mouth, and that perfectly *cold*, instead of alarming the patient by holding a red-hot iron before his face; it being at once raised to the required heat, and no more than the mere point of the wire used being heated; also being at once cooled on simply removing the finger from the spring."—*Boston Medical and Surgical Journal.*

*Tuberculous Cavern Injected.*—M. Rochat relates the case of a weaver, laboring under all the symtoms of phthisis, who had a fistula near the point of the scapula, which was found to communicate with a tuberculous cavern in the right lung. A more direct opening was made into the cavern by applications of caustic potash, near the edge of the third dorsal vertebra. Through this a large quantity of tuberculous matter was washed out, and into the cavern a tincture of iodine and iodide of potash injected. The patient was put upon cod-liver oil, and has entirely recovered.—*Western Lancet.*

*Magendie's Successor at the Academy of Sciences of Paris.*—The candidates for the membership vacant by the death of Magendie—were Messrs. Jobert (de Lamballe), Longet, Cruveilhier, Poiseuille, Baudens, and Langier. The successful competitor is M. Jobert (de Lamballe), surgeon to the Hotel Dieu. Those who ran close to him were M. Longet, and M. Cruveilhier.—*ib.*

*Chloroform in Pneumonia.*—A Hungarian physician, Dr. Stohandl, reports three cases of pneumonia in which much benefit was derived from the inhalation of small quantities of chloroform (30 to 40 drops), repeated several times a day. After each inhalation the symtoms were relieved; after four or six hours they again became aggravated, but were again relieved by a repetition of the inhalation. In from five to eight days a cure was effected.—*Revue de Therap.*, &c.

*Hydrophobia.*—In the course of Dr. Blatchford's paper on hydrophobia, read before the American Medical Association, at its last annual meeting, at Detroit, the following curious facts were reported as taking place in Prussia:—In 1810, there were in that kingdom 104 deaths from hydrophobia; in 1811, 117; in 1812, 101; in 1813, 85; in 1814, 127; in 1815, 79; in 1816, 201; in 1817, 228; in 1818, 260; in 1819, 356—making a total of 1,658 deaths in ten years, in Prussia alone. It is mentioned also as a curious fact, that in Cyprus and Egypt, hydrophobia has never been known to occur. It is believed also that the disease is incident to no particular month in the year, as statistics show, on the whole, as many deaths at one month of the year as at any other—there being no real difference between summer and winter. The Doctor believed the constitutional irascibility of the dog was the true etiology of canine madness, and that excision is the only means now known which affords any reasonable hope of successful prevention. The report pronounces as an utter fallacy the general idea that the dog star has anything to do with the origin of virus in the dog, or that summer has any special preponderance over winter in the existence of cases of hydrophobia. The facts submitted, and which had been collected by the Committee, show the following result: Out of 72 cases, 54 were bitten by dogs, 6 by cats, 1 by a raccoon, and 1 by a cow. Out of 62 cases, 4 died the first day, 9 the second, 6 the third, 18 the fourth, 4 the fifth, 2 on each the sixth, seventh and tenth days, and 1 on the twenty-first. The 22 bites occurred in March, April and May; 17 the next quarter; 18 the next, and 22 the last. The average of the time of sickness was 66 days, but this lengthy average was enhanced by two strongly marked cases, lasting 365 and 360 respectively. The usual average is 41 days.—*New York Times, May* 15.

*Plural Births.*—We know not whether the "baby-shows" have created a demand for human litters, but we have certainly remarked of late an unusual number of instances of three and four children at a birth. The following instance of a quintette is from the *London Lancet* of April 19th:

On Sunday morning the 13th inst., between the hours of 8 and 10, Mrs. E. Phin, wife of Edward Phin, a guard in the service of the London and North Western Railway Company, residing at 144 Scofield street, Bloomsbury, Birmingham, was safely delivered of five children—three boys born alive and doing well, and two girls born dead.

*Dentists Soldering Lamp.*—Mr. T. J. Haskell, dentist, of Philadelphia, has recently constructed a soldering lamp, possessing several advantages over those usually employed for the purpose. It will hold about a pint of alcohol; upon the top a piece of slate is let in for grinding borax, as well as a place for the borax and solder. To the back part of the lamp there is a cup for water and a match box at the side. Near the spout for the wick is, what we presume is intended for a ventilator or escape pipe for any steam which may be generated inside while soldering. We do not, however, perceive that this can be of any particular advantage, as this will pass off through the wick-spout as generated; besides, it seems to us, though we may be mistaken, never having used the lamp, that the flame might be communicated to the alcohol within through this tube. This, however, might be easily prevented by closing the tube. There is also a place on the side for pouring the alcohol into the lamp.—*American Journal.*

*A Valuable Bucket.*—Among the many modes of making money here, none, I think, surpasses the following: A surgeon told me he went one day into the tent of a brother medicus, on the Bendingo, just as a patient was going out, "I have been stopping a tooth," said the surgeon. "Do you get good cement here?" inquired my friend. "Admirable! I saw an old gutta percha bucket selling in a lot of tools one day at an auction. I bought the lot for the sake of the bucket, which cost me five shillings. I have already stopped some hundreds of teeth with the gutta percha at a guinea each, and shall no doubt, stop thousands with it before the old bucket is used up. It is a fortune to me. My name is up for an unrivalled dentist, and they come to me from far and near."—*Two Years in Victoria.*

*Dr. Beale's Reception.*—A reception has recently been given, by upwards of fifty members of the dental profession in New York, to Dr. Beale and his family, at the house of Dr. Brown, near Jones street, to congratulate him, after the act of liberation awarded to him by the Governor of Pennsylvania.—*Charleston Medical Journal.*

*Medical Science.*—The Sultan has authorized the formation of a Medical Society at Constantinople, and has sanctioned its title as the Imperial Medical Society of Constantinople. The new Institution was started by the English medical men at Scutari.

*Gestation Prolonged beyond the Natural Term by Homœopathic Means.*—The *Gazette Hebdomadaire* of Paris mentions, in a sarcastic strain, a little bit of Hahnemannian sorcery recently perpetrated in the French Capital. Puerperal fever having been rife of late, many ladies, on the eve of parturition, were in great alarm; one, however, expressed herself with great confidence on the subject, saying, that her homœopathic attendant was giving her certain globules, by means of which her confinement would not take place at the accustomed period, but *would be delayed* until the epidemic had abated.—*London Lancet.*

*Western Giants in their Slumber.*—The Burlington (Iowa) State Gazette says, that while some workmen were engaged in excavating for the cellar of Governor Grime's new building, on the corner of Maine and Valley streets, they came upon an arched vault some ten feet square, which, on being opened, was found to contain eight human skeletons of gigantic proportions. The walls of the vault were about fourteen inches thick, well laid up with cement or indestructible mortar. The vault is about six feet deep from the base to the arch. The skeletons are in a good state of preservation, and we venture to say are the largest human remains ever found, being a little over eight feet long.—*Calendar* (Hartford.)

*A Gold Filling Removed from the Neck.*—A lady in Tennessee, had a gold filling, about the size of a squirrel shot, recently removed from the side of her neck, immediately beneath the angle of the jaw. She was ignorant of the manner in which it got there, but it probably escaped from one of her teeth while eating and lodged in the fauces or a fold of the mucous membrane of the lower part of the mouth, and from thence made its way to the place from which it was removed.—*Ibid.*

*Professor Oppolzer at Warsaw.*—Professor Oppolzer has been sent from Vienna to Warsaw, in order to treat Prince Paskiewitch, who, notwithstanding he is an enemy to all doctors, was so taken with the Professor that he desired him to prolong his stay. Oppolzer has diagnosed a perforating ulcer of the stomach, and the prognosis is rendered still worse by the fact of the existence of a large, painful carbuncle in the spinal region. The Professor, it is said, receives 2,400 francs per diem, all the expenses of his journey and residence being defrayed. Moreover, crowds of the inhabitants of Warsaw consult him.—*Lancet.*

*A Fortunate Obstetrition.*—M. Paul Dubois, who officiated as accoucheur to the Empress Eugene, has been promoted by his Imperial master to the grade of Commander of the Legion of Honor, in consequence of the happy delivery of the Empress of a live Prince. He also received a fee of one hundred thousand francs, which in this utilitarian age is worth more than all the honors of Knighthood combined.—*St. Louis Medical and Surgical Journal.*

*Two Children Born with an interval of Nine Months and ten days.*—Dr. George W. Lewis, of Loydeville, Clark county, Kentucky, reports, that, on the 15th of April, 1853, he attended a woman in labor, and delivered her of a small weakly child, which died in the course of a few hours. Again, on the 25th of January, 1854, he attended the same woman in labor, and delivered her of a stout, healthy child, apparently born at full time, and who did well. The period between her two accouchements was exactly nine months and ten days.—*Ibid.*

*Muriate of Morphia and Coffee in Neuralgia.*—M. Boileau reports that he has derived great relief in the paroxysms of neuralgia, from the administration of the muriate of morphia in a very hot infusion of highly roasted coffee. The dose is one centigramme (one-seventh grain) for an adult, and less in other ages and in peculiar temperaments. This may be repeated when a violent paroxysm recurs, and if necessary it may be increased by frictions ; but M. Boileau has never gone beyond two centigrammes.—*Med. Times and Gazette.*

*Apothecaries' Charges after Midnight.*—A correspondent sends us his complaint of an apothecary who would not let him have some medicine after midnight, because he had not with him the necessary change—a sum which was a great deal larger than he had often paid by daylight. The case may have been aggravated. But there is certainly no sin in charging more for getting up out of a sound sleep, opening a store and preparing a medicine at an unseasonable hour, than for composing the same package by daylight. We suspect that all wise apothecaries are careful to make this proper distinction in their charges. If they do not, they ought to, in self-defense.—*New York Daily Times.*

